# Bilateral antrochoanal polyps in an adult

**DOI:** 10.1590/S1808-86942011000400023

**Published:** 2015-10-19

**Authors:** David Weber Sampaio Sousa, Sebastião Diógenes Pinheiro, Viviane Carvalho da Silva, João Paulo Catunda Bastos

**Affiliations:** 1 Medical doctor, Ceará Federal University. Medical resident in the Otorhinolaryngology Unit of the Walter Cantídio University Hospital, Medical School, Ceará Federal University; 2 Medical doctor, São Paulo University. Associate professor and head of the Otorhinolaryngology Unit of the Walter Cantídio University Hospital, Medical School, Ceará Federal University; 3 Master degree in community health, Medical School, Ceará Federal University. Assintant physician of the Otorhinolaryngology Unit, Medical School, Ceará Federal University; 4 Medical doctor, Ceará Fedral University. Medical resident in the Otorhinolaryngology Unit of the Walter Cantídio University Hospital, Medical School, Ceará Federal University

**Keywords:** endoscopy, nasal obstruction, nasal polyps

## INTRODUCTION

Antrochoanal polyps (ACPs) are single benign polypoid tumors that originate in the mucosa of the maxillary sinus; they may cross its ostium and extend to the floor of the nose, reaching the choana and nasopharynx.^1^ ACPs are more common in males before age 40 years, mostly children, teenagers, and young adults.^1^, ^2^, ^3^, ^4^, ^5^, ^6^

ACPs are almost always unilateral; there are few published cases of bilateral ACPs in the international literature.^2^, ^3^, ^4^, ^5^, ^6^ This paper presents bilateral ACP case in an adult.

## CASE REPORT

A male patient aged 37 years presented with complaints of progressive nasal block for the past four years. He denied other nasal symptoms such as rhinorrhea, sternutation crises, itching, hyposmia, or pain. The nasal block improved partially when the patient used topical decongestants; he was asked to stop this medication on the first visit. The patient reported no otologic or pharyngolaryngeal symptoms, asthma, or salicilate intolerance.

Nasofibroscopy revealed a polypoid tumor emerging from each maxillary sinus through widened ostia and extending to the nasopharynx.

**Figure 1:**
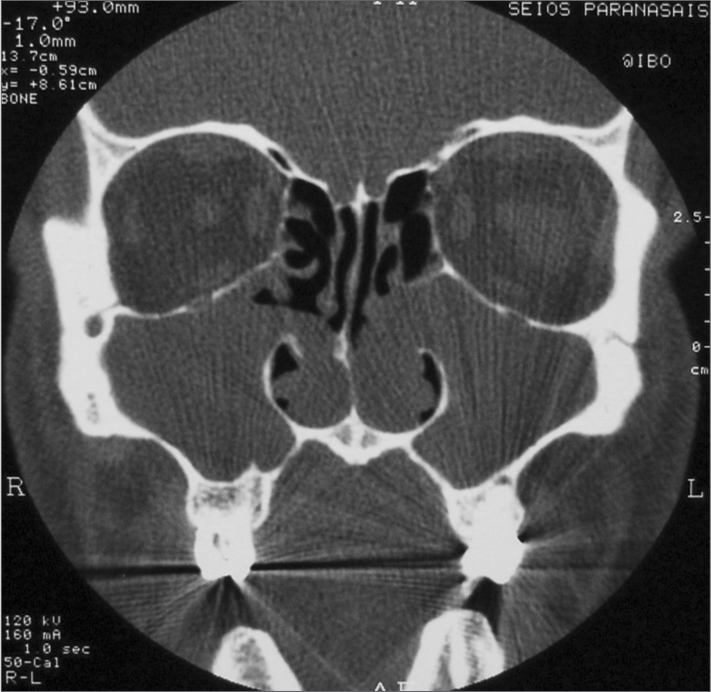
CT of the paranasal sinuses - coronal section showing tumors in both maxillary sinuses, which extend to the nasal cavity through the middle meati.

Computed tomography of the paranasal sinuses showed that the maxillary sinuses were filled with soft tissue density material that reached the nasal cavity and the choanae. The other sinuses were normally aerated (Fig. 1).

The tumors were removed surgically - maxillary antrostomy (Caldwell-Luc) with a nasal endoscopy technique. The maxillary sinuses were each filled with a single cystic tumor that was implanted on the lateral wall.

Histology of the two lesions described them as inflammatory polyps. The patient had no postoperative events. The tumors have not recurred six months after the procedure.

## DISCUSSION

Gustav Killian first described ACPs in 1906; these tumors comprise about 4% to 6% of all nasal polyps in the general population, and 28% to 33% in children.^1^, ^2^, ^3^, ^4^, ^5^, ^6^ Bilateral ACPs are rare; seven cases have been reported in the English scientific literature by April 2010, two of which in adults.^2^, ^3^, ^4^, ^5^, ^6^

The etiology of ACPs is unclear.^3^, ^4^, ^5^, ^6^ Chronic rhinosinusitis, cystic fibrosis, and allergy have been often implicated.^1^, ^2^, ^3^, ^4^, ^5^, ^6^ Studies have shown that ACPs usually originate from the lateral wall or the floor of the maxillary sinus.^3,^^6^ These tumors are histologically indistinguishable from intramural cysts in their sinus portion.^1,^^2^ Berg et al. (1988) have suggested that ACPs develop from intramural cysts in the maxillary sinus.^2,^^6^ This hypothesis does not explain the rarity of bilateral ACPs, especially because intramural cysts are often bilateral.^5^

The most common symptom of ACPs is unilateral or bilateral nasal block.^1,^^5^ Other findings include snoring, sleep apnea, oral breathing, rhinorrhea with pus, postnasal discharge, epistaxis, dyspnea, hyposmia, dysphagia, and weight loss.^1,^^2^ Depending on their volume, ACPs may obstruct the Eustachian's tube and cause secretory otitis media.^1^

The differential diagnosis should be made with retention mucous cysts, mucoceles, maxillary rhinosinusitis, meningoencephalocele, olfactory stesioneuroblastoma, angiofibroma, and inverted papilloma.^1,^^2^

Computed tomography and nasofibroscopy are the gold standard tests for diagnosing ACPs.^1,^^2^

Surgical treatment is mandatory.^1,^^2,^^5^ Recurrences are rare when the Caldwell-Luc approach is used; this method provides an ample view and complete removal of involved sinus tissues.^2^, ^3^, ^4^, ^5^ Surgeons tend to avoid this procedure in children aged below 8 years because of the risk of injuring anterior dental roots and maxillary growth centers.^2^, ^3^, ^4^, ^5^ In this age group, tumors are removed by avulsion, which has a high recurrence rate.^4,^^5^

Another possible approach is nasal endoscopy. Its advocates highlight the lower recurrence and complication risks of this approach.^2^, ^3^, ^4^, ^5^

## FINAL COMMENTS

Bilateral ACPs are rare; the cases reported thus far have not led to a plausible explanation for this tumor. Studies defining the approach of choice for treating this disease are sparse in the literature.
